# *Candida* colonization of the esophagus and gastric mucosa; a comparison of patients taking proton pump inhibitors and those taking histamine receptor antagonist drugs 

**Published:** 2021

**Authors:** Batoul Mottaghi, Mohammad Hassan Emami, Padideh Riahi, Alireza Fahim, Hojjatolah Rahimi, Rasoul Mohammadi

**Affiliations:** 1 *Department of Medical Parasitology and Mycology, School of Medicine, Isfahan University of Medical Sciences, Isfahan, Iran*; 2 ^*2 *^ *Poursina Hakim Digestive Diseases Research Center, Isfahan University of Medical Sciences, Isfahan, Iran*; 3 ^*3 *^ *Infectious Diseases and Tropical Medicine Research Center, Isfahan University of Medical Sciences, Isfahan, Iran*

**Keywords:** Candida colonization, Gastroesophageal, Proton pump inhibitors, Histamine receptor antagonist

## Abstract

**Aim:**

Investigation of *Candida* colonization of the esophagus and gastric mucosa in patients taking proton pump inhibitors in comparison with those taking histamine-2 receptor antagonists.

**Background:**

*Candida* species are normal flora of alimentary tract that can cause infection of the esophagus and gastro-intestinal tract in immunocompromised patients. Consumption of proton pump inhibitors and histamine-2 receptor antagonists has been shown to alter the gastric pH, which may predispose the esophagus and stomach to floral transmission and colonization

**Methods:**

Two hundred and forty-five clinical specimens were obtained from 91 patients who underwent endoscopy from September 2019 to February 2020. Direct microscopy with KOH 10% and subculture on sabouraud dextrose agar containing chloramphenicol was used as primary screening. PCR with ITS primers was performed to amplify the ITS1-5.8SrDNA-ITS2 region, and the *Msp*I restriction enzyme was used for RFLP to identify clinical isolates.

**Results:**

Seventy cultures out of 245 specimens were positive for *Candida* colonization (28.5%). Colonization of *Candida* species in gastric acid and gastric tissue biopsies of patients who took PPIs and H2 blockers was significantly higher than in those in the control group (*p= 0.001*). The use of ranitidine, pantoprazole, and omeprazole increased the risk of gastric candidiasis by 10.60, 9.20, and 12.99 times, respectively (*p< 0.05)*.

**Conclusion:**

The use of PPIs and H2 blockers, ageing, and consumption of vegetables were main risk factors for gastric colonization in the present survey; other variables, such as *Candida* species, cigarette smoking, and alcohol consumption, were not implicated in the development of gastroesophageal lesions. Further investigations are necessary to understand how these predisposing factors change the host’s defense mechanisms and increase colonization of fungi at mucosal surfaces.

## Introduction

 The genus *Candida* is a normal flora of the alimentary tract. *Candida* infection of the esophagus and gastro-intestinal (GI) tract typically occurs in immunocompromised hosts (1). In this population, the esophagus is the most routinely involved organ, followed by the stomach and small bowel (2). A number of predisposing factors favorable to fungal infections are diabetes, malnutrition, old age, burns, parenteral nutrition, trauma, surgical operations, steroidotherapy, bladder or intravascular catheterization, H2-blocker therapy, immunosuppressive treatment, and recurrent antibiotic use (3). Patients who have three or more predisposing factors are considered to be at high risk for fungal infection (4). *Candida *esophagitis and gastritis have been reported to be increasing in those consuming acid suppressing therapy (AST) (5). AST, proton pump inhibitors (PPIs), and histamine-2 receptor antagonists (H2RAs) have been shown to be the most useful drugs in patients with gastro-esophageal reflux disease (GERD). These drugs are favorably efficient in treating acid-mediated disorders of the upper digestive tract such as peptic ulcers and acute nonvariceal bleeding, and also for preventing nonsteroidal anti-inflammatory drug (NSAID)-related injury and stress ulcers (6-8). PPIs are also prescribed in combination with antibacterials for *Helicobacter pylori *eradication; however, they alter the gastric pH, which may predispose a patient to floral transmission and colonization of the esophagus and stomach by oral yeasts (9). In this study, *Candida* colonization of the esophagus and gastric mucosa was compared in patients taking PPIs and those taking H2RAs. 

## Methods

The investigation protocol was reviewed and approved by the Ethics Committee of Isfahan University of Medical Sciences (IR.MUI.MED.REC.1398.236). The informed consent form was completed and signed by each patient.

From September 2019 to February 2020, 245 clinical samples were obtained from 91 patients who underwent endoscopy in Poursina Hakim Digestive Diseases Research Center, Isfahan University of Medical Sciences, Isfahan, Iran. Clinical samples were gastric juices (n=89), gastric tissue biopsies (n=77), and esophageal biopsy specimens (n=79). 

**Inclusion criteria:** Patients who had taken PPIs or H2RAs drugs.

**Exclusion criteria:** Patients who had taken antifungal drugs for the prior 7 days and those taking PPIs and H2RAs drugs simultaneously.

In the experimental group, 22, 18, 11, and 6 patients had taken omeprazole, pantoprazole, ranitidine (H2RAs), and other PPIs (such as: rabeprazole, lansoprazole, and nolpaza), respectively. The control group consisted of 34 patients who had not taken PPIs or H2RAs. 

**Phenotypic Tests**

Direct microscopy was applied for each specimen using potassium hydroxide (KOH) 10%. Sabouraud dextrose agar (SDA) (Biolife, Italy) with chloramphenicol (Merck, Germany) (0.04 g/L) used for culture. The specimens were incubated at 37 °C and evaluated for fungal growth up to 4 days.

**Molecular Identification**

**Polymerase chain reaction (PCR)**

Genomic DNA from the clinical strains was extracted using the boiling method (10). Briefly, a loopful of fresh colonies was suspended in 80 µL of double distilled water and boiled for 20 minutes, then centrifuged for 6 minutes at 5000 rpm. The supernatant containing DNA was kept for PCR. The PCR mixture, comprising 5 μL of 10× reaction buffer, 0.4 mM dNTPs, 1.5 mM MgCl2, 30 pmol of ITS1 primer (5ʹ-TCC GTA GGT GAA CCT GCG G-3ʹ), 30 pmol of ITS4 primer (5ʹ-TCC GCT TAT TGA TAT GC-3ʹ), 2.5 U of Taq polymerase, and 3 μL of extracted DNA, was applied in a final volume of 50 μL. The PCR cycling conditions were an initial denaturation phase at 95 °C for 5 min, followed by 30 cycles of denaturation at 95 °C for 1 min, annealing at 54 °C for 50 s, extension at 72 °C for 1 min, and a final extension phase at 72 °C for 7 min. Five microliters of each PCR amplicon were run on 1.5% agarose gel, stained with 0.5 μg/mL ethidium bromide, visualized by gel documentation system (UVITEC, UK), and then photographed.

**Restriction fragment length polymorphism (RFLP)**

PCR products were digested in a final volume of 15 μL containing 3 μL double distilled water, 1 U of *Msp*I restriction enzyme (Fermentas, Vilnius, Lithuania), 1.5 μL buffer, and 10 μL PCR product at 37 °C for 2 hours. RFLP products were run on 2% agarose gel, stained with 0.5 μg/mL ethidium bromide, visualized by gel documentation system (UVITEC, UK), and then photographed.

**Data Analysis**

The variables were analyzed by chi-squared, independent sample t-test, and logistic regression in the SPSS version 23 (Armonk, NY: IBM Corp). The cut-off point for statistical significance was set at *p* < 0.05. 

## Results

**Figure 1:**
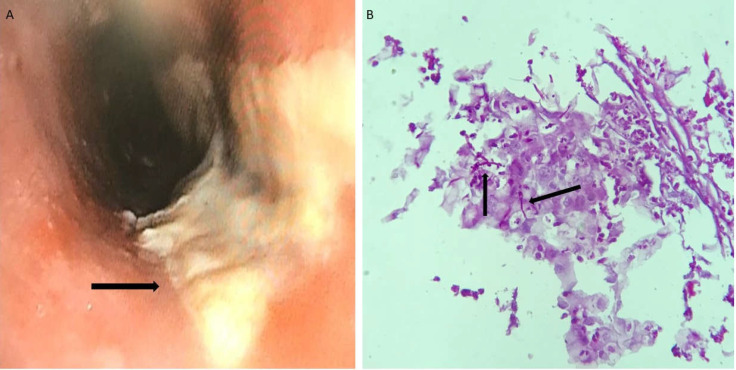
**A.** Endoscopic finding of esophageal candidiasis; multiple thick and whitish plaques (black arrow); **B:** Presence of *Candida* (pseudo) hyphae (black arrows) in a smear of esophageal biopsy stained by the PAS-method (original magnification: ×40).

**Figure 2:**
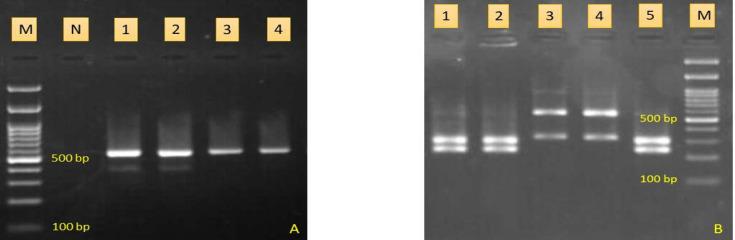
**A.** PCR products of four Candida species. Lane 1 is C. parapsilosis, lane 2 is C. tropicalis, lanes 3, 4 are C. albicans, lane N is negative control, and lane M is molecular size marker; **B:** Agarose gel electrophoresis of RFLP products. Lanes 1, 2, 5 are C. albicans, lanes 3, 4 are C. glabrata, and M is 100 bp DNA size marker

Seventy cultures out of 245 specimens were positive for *Candida* colonization (28.5%) (Figure 1). Male-to-female ratio was 40/51. The age of patients ranged between 21 and 85 years, with a median age of 52.8 years. The most common predisTposing factors were antibiotic consumption (8.8%), diabetes (6.6%), cancer (6.6%), hypothyroidism (5.5%), hyperthyroidism (2.2%), and gastric bypass surgery (2.2%). Cefixime (n=3), cephalexin (n=2), penicillin (n=2), and ciprofloxacin (n=1) were used as antibiotic therapies for patients. The majority of patients suffered from gastritis (34%), dysphagia (26.4%), flatulence (19.8%), and acid reflux (19.8%). The pH of gastric acid of patients was 1 (n=34), 2 (n=10), 3 (n=8), 4 (n=5), 5 (n=3), 6 (n=4), 7 (n=14), 8 (n=12), and 12 (n=1). All isolates were identified by PCR-RFLP technique (Figure 2). *Candida albicans* was the most prevalent *Candida* species (n=50, 71.4%), followed by *C. glabrata* (n=15, 21.4%), *C. parapsilosis* (n=2, 2.8%), *C. famata* (n=2, 2.8%), and *C. tropicalis* (n=1, 1.4%). Colonization of *Candida* species in the gastric acid of patients who took PPIs and H2 blockers was significantly higher than those in the control group (*p = 0.001*). The average time of drug use in the case group was 12 months ± 26 days. *Candida* colonization in gastric tissue biopsies between PPIs and H2 blocker consumers and the control group was statistically significant (*p = 0.043*); however, it was not significant between *Candida albicans *and non-*albicans Candida* species (*p > 0.05*). There was an association among *Candida* colonization, ageing, and consumption of vegetables (*p < 0.05*). Ageing and use of vegetables increased the risk of colonization in gastric juices by 1.04 and 1.01 times, respectively. Furthermore, the use of ranitidine, pantoprazole, and omeprazole increased the chances of gastric candidiasis by 10.60, 9.20, and 12.99 times, respectively (*p < 0.05*). Regression analysis detected that colonization of *Candida* spp. in the esophagus of patients who took PPIs and H2 blockers was not significantly higher than in those who were in control group (*p > 0.05*).

## Discussion

Gastroesophageal candidiasis has been reported in 16% of patients with mild gastric ulcers, 20% of those with gastric cancer, and 27% of those with esophageal cancer (11). The gastrointestinal microbiome could play a substantial role in the pathogenesis of some immune-mediated intestinal conditions. Gastric acid is a main barrier for the inhibition of fungal and bacterial overgrowth of the stomach and small bowel (12). AST consumption has been reported to be connected to an increased incidence of *Candida *infections such as* Candida *esophagitis (CE) and gastric candidiasis (5, 13). CE is currently the most prevalent esophageal microbial infection among patients with human immunodeficiency virus (HIV), and its frequency in HIV (-) patients has been reported to be increasing (14). None of the patients in the present study were HIV (+). CE is found in 20% of healthy individuals and in 24-66% of patients using inhaled steroids (15, 16). In accordance with the current findings, *Candida albicans *is the most prevalent etiologic agent; however, some species including *C. krusei, C. glabrata, C. tropicalis*, and *C. parapsilosis *have also been implicated (17). The main mechanism of AST connected to demolition of the gastric acid barrier is associated with gastritis because of *Candida *colonization in the esophagus and gastric mucosa (5, 18). There are two types of yeast in gastric mucosa: 1) as a true microflora of gastric environment (19), and 2) as a transient colonizer of gastric epithelium (20). In general, it seems that both groups are well-adapted to establish in the environment of the gastric mucosa. PPIs have been revealed to be the most useful drugs in patients with gastro-esophageal reflux disease; however, they may boost the survival of microorganisms by elevating the gastric pH. In this regard, Husebye et al. (21) showed that an increment of one pH unit correlated with an increase of about 14% in microbial counts in the stomach and small intestine. In the present study, an approximately 9% increase in the colony count in the stomach with an increment of one pH unit was observed. Buhling et al. showed a transient increment of *Candida* species in the intestine in 50% of patients promptly after treatment with omeprazole (22). Karmeli et al. (23) showed a significantly rise in *Candida *colonization in gastric mucosa 30 days after beginning omeprazole therapy, while Goscimski et al. (24) reported similar findings following one week of treatment with pantoprazole. In the current study, 33.3% and 27.7% of patients showed *Candida *colonization 7 days after treatment with pantoprazole and omeprazole, respectively. Extended use of PPIs can cause hypergastrinemia and hypochlorhydria, food allergy, mineral and vitamin deficiencies, increased risk of infections, and drug interactions (25, 26). They have also been reported to suppress anti-*Candida *macrophage activity (27). One of the main limitations of this study was the lack of immunological information of patients. PPI-induced gastric acid decrement can develop the microbial invasion by changing the gastrointestinal microbiome, reducing gastric mucus viscosity, and suppressing leucocyte activity (13, 28). PPIs might enhance *Candida* colonization by inhibiting the growth of *Lactobacillus* species as normal flora. By producing lactic acid, *Lactobacillus* decreases the quantity of *Candida* species. Therefore, the inhibition of *Lactobacillus* population by PPIs could lead to *Candida* colonization and infection (29). Another limitation of the current investigation was the lack of data for bacterial species and fungal-bacterial endosymbiosis. Gastric acid suppression is considered an important risk factor for nosocomial infections, particularly in patients with defective immune systems and those using indwelling catheters (30). Moreover, the use of H2RAs has been linked to gastric acid suppression and increased gastrointestinal tract colonization. Although the consumption of H2RAs can increase the risk of enterocolitis, sepsis and meningitis, and bloodstream infections with the genus *Candida *(31-33), no candidemia or systemic candidiasis cases were seen in the present investigation. Ross et al. (34) showed that H2RAs decreased gastric acidity less than that induced by PPIs; nevertheless, 60% of patients in the current study who took ranitidine had gastric acid pH levels ≤ 2. *Candida* colonization in the oral cavity is potentially considered for gastroesophageal candidiasis, especially in immunocompromised patients. It is impressed by the oral bacterial populations and immune system of the host (35, 36). Only 4.3% of patients in the present study were found to have oral candidiasis. It was proposed that *Candida* species change phenotypically to hyphae when colonizing human and animal tissues (Figure 1B). Controversially, some reports have indicated that most dimorphic yeasts can invade tissues by budding form (37). Since *C. glabrata* cannot produce hyphae or pseudohyphae, all *C. glabrata* isolates (n=15) showed budding form in tissues in the present study. The correlation between *Candida* colonization and diabetes mellitus has also been controversial. Many investigations have revealed severe colonization of *Candida* spp. in patients with diabetes mellitus in comparison to non-diabetic patients (38, 39); however, other research has not showed a remarkable increase in *Candida* in diabetic patients (40, 41). In the present study, 6.6% of patients had diabetes mellitus with mild colonization. The extent of *Candida* growth was classified as none (0 colony), a few (<10 colonies), mild (10 to 100 colonies), or severe (>100 colonies) (42). 

Antibiotic consumption can provoke *Candida* colonization, particularly in those admitted to an intensive care unit (43). According to this risk factor, however, *Candida* colonization in esophagus and gastric tissue biopsies between consumers of PPIs and H2 blockers and the control group was not statistically significant (*p > 0.05*). The most prevalent *Candida* species isolated in the current investigation was *albicans*, which is consistent with Delisle et al. (44) and Garbino et al. (45). *Candida glabrata* was the most frequently isolated non-*albicans Candida* in the current study. This differs from surveys by Amiri et al. (43) and El-Ebiary et al. (46) who reported *C. tropicalis* and *C. krusei*, respectively, as the most prevalent non-*albicans Candida* species; however, it is consistent with Garbino et al. (45). Some investigations have found an association between *Candida* colonization and invasion in mucosa and increased hospital mortality (44, 47). No information about the mortality rate of patients in the current investigation was available. 

It was concluded that the use of PPIs and H2 blockers, ageing, and consumption of vegetables were main risk factors for gastric colonization in the present survey, and *Candida* species, cigarette smoking, alcohol consumption, gender, age, meat and bread consumption were not implicated in the development of gastric lesions. By analysis of the variables using logistic regression, no significant factor for the incidence of candidiasis in esophageal samples was found. Further investigations are needed to understand how these predisposing factors change the host’s defense mechanisms and increase colonization of fungi at mucosal surfaces. This data can lead to more consideration related to chronic disorders, immune responses, and appropriate medications.

## References

[1] Goyal P, Bansal S, Kaur P, Goyal O (2016). Candida associated giant non-healing gastric ulcer in an immunocompetent host. J Gastroenterol Liver Dis.

[2] Tsukamoto H (1986). Clinicopathological studies on fungus infections of the digestive tract. Nihon Shokakibyo Gakkai Zasshi.

[3] Zwolińska-Wcisło M, Budak A, Bogdał J, Trojanowska D, Stachura J (2001). Fungal colonization of gastric mucosa and its clinical relevance. Med Sci Monit.

[4] Savino JA, Agarwal N, Wry P, Policastro A, Cerabona T, Austria L (1994). Routine prophylactic antifungal agents (clotrimazole, ketoconazole, and nystatin) in nontransplant/nonburned critically ill surgical and trauma patients. J Trauma.

[5] Daniell H (2016). Acid suppressing therapy as a risk factor for Candida esophagitis. Dis Esophagus.

[6] Alshamsi F, Belley-Cote E, Cook D, Almenawer SA, Alqahtani Z, Perri D (2016). Efficacy and safety of proton pump inhibitors for stress ulcer prophylaxis in critically ill patients: a systematic review and meta-analysis of randomized trials. Crit Care.

[7] Reimer C (2013). Safety of long-term PPI therapy. Best Pract Res Clin Gastroenterol.

[8] Safavi M, Sabourian R, Foroumadi A (2016). Treatment of Helicobacter pylori infection: Current and future insights. World J Clin Cases.

[9] Shah N, Cavanagh Y, Shulik O, Patel P, DeBari VA, Baddoura W (2015). Proton Pump Inhibitors and Corticosteroids as Synergistic Risk Factors for Candida Esophagitis. Am J Gastroenterol.

[10] Silva GAd, Bernardi TL, Schaker PDC, Menegotto M, Valente P (2012). Rapid yeast DNA extraction by boiling and freeze-thawing without using chemical reagents and DNA purification. Braz Arch Biol Technol.

[11] Scott B, Jenkins D (1982). Gastro-oesophageal candidiasis. Gut.

[12] Neal K, Logan R (2001). Potential gastrointestinal effects of long‐term acid suppression with proton pump inhibitors. Aliment Pharmacol Ther.

[13] Freedberg DE, Lamousé-Smith ES, Lightdale JR, Jin Z, Yang Y-X, Abrams JA (2015). Use of acid suppression medication is associated with risk for C difficile infection in infants and children: a population-based study. Clin Infect Dis.

[14] Mimidis K, Papadopoulos V, Margaritis V, Thomopoulos K, Gatopoulou A, Nikolopoulou V (2005). Predisposing factors and clinical symptoms in HIV‐negative patients with Candida oesophagitis: are they always present?. Int J Clin Pract.

[15] Mullaoglu S, Turktas H, Kokturk N, Tuncer C, Kalkanci A, Kustimur S (2007). Esophageal candidiasis and Candida colonization in asthma patients on inhaled steroids. Allergy Asthma Proc.

[16] Fidan F, Uslan İ, Çetinkaya Z, Sezer M, Kara Z, Ünlü M (2006). Prevalence of esophageal candidiasis in patients treated with ınhaled and short course systemic steroids. Med J Kocatepe.

[17] Naito Y, Yoshikawa T, Oyamada H, Tainaka K, Morita Y, Kogawa T (1988). Esophageal candidiasis. Gastroenterol Jpn.

[18] Jacobs C, Coss Adame E, Attaluri A, Valestin J, Rao SSC (2013). Dysmotility and proton pump inhibitor use are independent risk factors for small intestinal bacterial and/or fungal overgrowth. Aliment Pharmacol Ther.

[19] Von Rosenvinge EC, Song Y, White JR, Maddox C, Blanchard T, Fricke WF (2013). Immune status, antibiotic medication and pH are associated with changes in the stomach fluid microbiota. ISME J.

[20] Karczewska E, Wojtas I, Sito E, Trojanowska D, Budak A, Zwolinska-Wcislo M (2009). Assessment of co-existence of Helicobacter pylori and Candida fungi in diseases of the upper gastrointestinal tract. J Physiol Pharmacol.

[21] Husebye E, Skar V, Høverstad T, Iversen T, Melby K (1995). Abnormal intestinal motor patterns explain enteric colonization with gram-negative bacilli in late radiation enteropathy. Gastroenterology.

[22] Bühling A, Radun D, Müller W, Malfertheiner P (2001). Influence of anti‐Helicobacter triple‐therapy with metronidazole, omeprazole and clarithromycin on intestinal microflora. Aliment Pharmacol Ther.

[23] Karmeli Y, Stalnikowitz R, Eliakim R, Rahav G (1995). Conventional dose of omeprazole alters gastric flora. Dig Dis Sci.

[24] Gościmski A, Matras J, Wallner G (2002). Microflora of gastric juice in patients after eradication of Helicobacter pylori and treatment with a proton pump inhibitor. Wiadomosci Lekarskie.

[25] Safe M, Chan WH, Leach ST, Sutton L, Lui K, Krishnan U (2016). Widespread use of gastric acid inhibitors in infants: are they needed? Are they safe?. World J Gastrointest Pharmacol Ther.

[26] De Bruyne P, Ito S (2018). Toxicity of long-term use of proton pump inhibitors in children. Arch Dis Child.

[27] Motegi H, Abe S, Tansho S, Suzuki D, Yamaguchi H, Hoshino E (2001). Suppressive effect of lansoprazole on anti-Candida activity of murine macrophages. Kansenshogaku Zasshi.

[28] Imhann F, Bonder MJ, Vila AV, Fu J, Mujagic Z, Vork L (2016). Proton pump inhibitors affect the gut microbiome. Gut.

[29] Wang K, Lin HJ, Perng CL, Tseng GY, Yu KW, Chang FY (2004). The effect of H2-receptor antagonist and proton pump inhibitor on microbial proliferation in the stomach. Hepatogastroenterology.

[30] Cohen S, Bueno de Mesquita M, Mimouni FB (2015). Adverse effects reported in the use of gastroesophageal reflux disease treatments in children: a 10 years literature review. Br J Clin Pharmacol.

[31] Guillet R, Stoll BJ, Cotten CM, Gantz M, McDonald S, Poole WK (2006). Association of H2-blocker therapy and higher incidence of necrotizing enterocolitis in very low birth weight infants. Pediatrics.

[32] Stoll BJ, Temprosa M, Tyson JE, Papile L-A, Wright LL, Bauer CR (1999). Dexamethasone therapy increases infection in very low birth weight infants. Pediatrics.

[33] Saiman L, Ludington E, Pfaller M, Rangel-Frausto S, Wiblin TR, Dawson J (2000). Risk factors for candidemia in neonatal intensive care unit patients. Pediatr Infect Dis J.

[34] Ross AL, Slain D, Cumpston A, Bryant AM, Hamadani M, Craig M (2012). Evaluation of an alternative posaconazole prophylaxis regimen in haematological malignancy patients receiving concomitant stress ulcer prophylaxis. Int J Antimicrob Agents.

[35] Sasaki K (2012). Candida-associated gastric ulcer relapsing in a different position with a different appearance. World J Gastroenterol.

[36] Soysa N, Samaranayake L, Ellepola A (2008). Antimicrobials as a contributory factor in oral candidiasis– a brief overview. Oral Dis.

[37] Gow NA, Brown AJ, Odds FC (2002). Fungal morphogenesis and host invasion. Curr Opin Microbiol.

[38] Belazi M, Velegraki A, Fleva A, Gidarakou I, Papanaum L, Baka D (2005). Candidal overgrowth in diabetic patients: potential predisposing factors. Mycoses.

[39] Kumar B, Padshetty N, Bai K, Rao M (2005). Prevalence of Candida in the oral cavity of diabetic subjects. J Assoc Phys India.

[40] Daniluk T, Tokajuk G, Stokowska W, Fiedoruk K, Sciepuk M, Zaremba M (2006). Occurrence rate of oral Candida albicans in denture wearer patients. Adv Med Sci.

[41] Kadir T, Pisiriciler R, Akyüz S, Yarat A, Emekli N, Ipbüker A (2002). Mycological and cytological examination of oral candidal carriage in diabetic patients and non‐diabetic control subjects: thorough analysis of local aetiologic and systemic factors. J Oral Rehabil.

[42] Lau AF, Kabir M, Chen SC-A, Playford EG, Marriott DJ, Jones M (2015). Candida colonization as a risk marker for invasive candidiasis in mixed medical-surgical intensive care units: development and evaluation of a simple, standard protocol. J Clin Microbiol.

[43] Amiri HM, Frandah W, Colmer-Hamood J, Raj R, Nugent K (2012). Risk factors of Candida colonization in the oropharynx of patients admitted to an intensive care unit. J Mycol Med.

[44] Delisle M-S, Williamson DR, Perreault MM, Albert M, Jiang X, Heyland DK (2008). The clinical significance of Candida colonization of respiratory tract secretions in critically ill patients. J Crit Care.

[45] Garbino J, Lew DP, Romand J-A, Hugonnet S, Auckenthaler R, Pittet D (2002). Prevention of severe Candida infections in nonneutropenic, high-risk, critically ill patients: a randomized, double-blind, placebo-controlled trial in patients treated by selective digestive decontamination. Intensive Care Med.

[46] El-Ebiary M, Torres A, Fabregas N, de la Bellacasa JP, Gonzalez J, Ramirez J (1997). Significance of the isolation of Candida species from respiratory samples in critically ill, non-neutropenic patients: an immediate postmortem histologic study. Am J Respir Crit.

[47] Olaechea P, Palomar M, León-Gil Ct, Alvarez-Lerma F, Jorda R, Nolla-Salas J (2004). Economic impact of Candida colonization and Candida infection in the critically ill patient. Eur J Clin Microbiol.

